# A novel orally available Syk/Src/Jak2 inhibitor, SKLB-850, showed potent anti-tumor activities in B cell lymphoma (BCL) models

**DOI:** 10.18632/oncotarget.22847

**Published:** 2017-12-01

**Authors:** Nannan Zhang, Guo Zhang, Ning Liu, Wanting Lin, Sen Ji, Mingwu Zheng, Kai Chen, Xiao Liang, Guobo Li, Yu Ma, Jun Zhu, Ting Niu, Lin-Li Li, Jiong Li, Yu-Quan Wei, Sheng-Yong Yang

**Affiliations:** ^1^ National Center for Birth Defect Monitoring, Key Laboratory of Obstetric & Gynecologic and Pediatric Diseases and Birth Defects, Ministry of Education, West China Second University Hospital, Sichuan University and Collaborative Innovation Center for Biotherapy, Chengdu, Sichuan, 610041, China; ^2^ Department of Hematology & Research Laboratory of Hematology, West China Hospital, Sichuan University, Chengdu, Sichuan, 610041, China; ^3^ Key Laboratory of Drug Targeting and Drug Delivery System of Ministry of Education, West China School of Pharmacy, Sichuan University, Chengdu, Sichuan, 610041, China; ^4^ Department of Obstetric & Gynecologic, West China Second University Hospital, Sichuan University, Chengdu, Sichuan, 610041, China

**Keywords:** SKLB-850, B cell lymphoma, multikinase inhibitor

## Abstract

B cell lymphoma (BCL) is the most frequently diagnosed type of non-Hodgkin lymphoma (NHL), and accounts for about 4% of all cancers in the USA. Kinases spleen tyrosine kinase (Syk), Src, and Janus kinase 2 (JAK2) have been thought as potential targets for the treatment of BCL. We have recently developed a multikinase inhibitor, SKLB-850, which potently inhibits Syk, Src, and JAK2. The aim of this study is to investigate the anti-BCL activities and mechanisms of action of SKLB-850 both in *vitro* and in *vivo*. Our results showed that SKLB-850 significantly inhibited BCL cell proliferation, and induced apoptosis of BCL cells. It could considerably decrease the secretion of chemokines CCL3, CCL4, and CXCL12. Oral administration of SKLB-850 considerably suppressed the tumor growth in BCL xenograft models (Ramos and HBL-1) in a dose-dependent manner. Immunohistochemistry of tumor tissues showed that SKLB-850 efficiently inhibited the activation of Syk/ERK, Src/FAK and JAK2/Stat3 pathways. Collectively, SKLB-850 could be a promising agent for the treatment of BCL, hence deserving further study.

## INTRODUCTION

B cell lymphoma (BCL) is one of the most frequently diagnosed lymphomas, which has been experiencing a substantial increase in incidence and mortality worldwide [1]. Chemotherapy had long been the only systematic treatment of BCL until the first monoclonal antibody (mAb) drug, rituximab, was approved by the US food and drug administration (FDA) to treat BCL in 1997 [2]. In 2013, a small molecule targeted drug, Ibrutinib [3], which is a BTK inhibitor, was also approved by US FDA for the therapy of BCL. Nevertheless, the BCL treatment still faces some challenges. For example, 30% - 50% of patients succumb to the BCL yet [4]. Rituximab has been reported to exert certain acute side effects such as acute infusion reasons, immune reconstitution defects, various infections, reactivation of hepatitis, intestinal perforation, and so on [5, 6]. Moreover, drug resistant mutations have also been found in BTK, leading that patients originally responding to the Ibrutinib treatment have no response anymore [7, 8]. Therefore, novel targeted agents for the BCL treatment are urgently demanded at present.

BCL has been demonstrated to be a complicated disease with multiple genes dysregulated. Among these genes, of particular importance are Syk, Src, and JAK2, in addition to BTK. Syk is a non-receptor tyrosine kinase, which could be recruited by dually phosphorylated ITAMs on Igα and Igβ after BCR (B cell receptor, BCR) aggregation. Syk phosphorylates several signal intermediates like BTK and BLNK, which then activate downstream signal pathways, including extracellular signal-related kinase (ERK) and NF-κB [9, 10]. Syk has been demonstrated to be critical for the survival and maintenance of malignant B cells [11, 12], and targeted inhibition of Syk has also been shown to be able to abrogate BCR signaling and induce apoptosis of BCL cells [13–15]. Src is also a non-receptor tyrosine kinase, and plays important roles in a variety of cellular signal transduction pathways [16]. It has been demonstrated to be involved in regulating tumor cell division, motility, adhesion, angiogenesis, and survival [17, 18]. Recent studies have indicated that activation of Src is highly related to the early stage phenotype of BCL and tumor growth [19]. These results highlight Src as a potential target for BCL therapy [18, 20]. JAK2, which is again a non-receptor tyrosine kinase, plays essential roles in transmitting signals from multiple cytokine receptors, and constitutive activation of JAK2 has been demonstrated to result in myeloproliferative neoplasms [21]. Recent studies further showed that mutation of JAK2 was capable of inducing BCL [22, 23, 24], and inhibition of JAK2 decreased IgM-induced STAT3 phosphorylation and increased apoptosis of tumor cells in a dose-dependent manner, which suggest that JAK2 could be a target for the treatment of BCL [22, 25].

**Figure 1:**
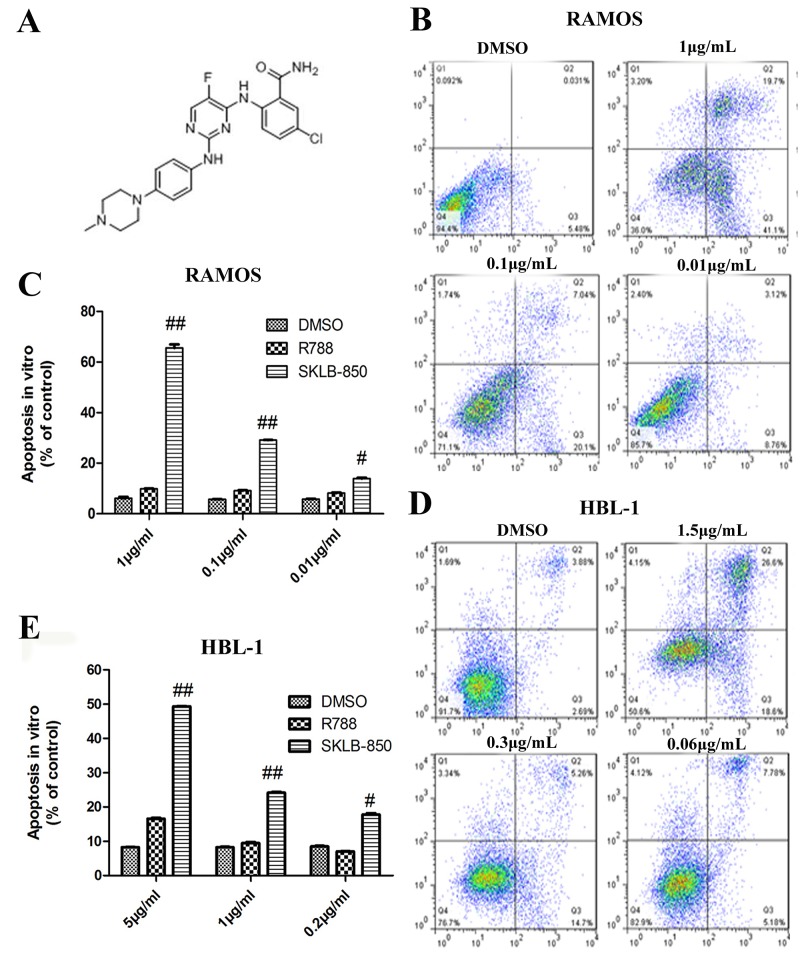
The chemical structure of SKLB-850 and its anti-tumor effects *in vitro* **(A)** The chemical structure of SKLB-850. **(B and C)** Apoptosis of Ramos cells induced by SKLB-850. Cells were treated by vehicle, R788 or indicated concentrations of compounds. After 18 hours, Cell were harvested and stained with AnnexinV-FITC and propidium iodide, then the apoptosis cells were analyzed by FCM. **(D and E)** Apoptosis of HBL-1 cells induced by SKLB-850. Cells were treated by vehicle, R788 or indicated concentrations of compounds. After 24 hours, Cell were harvested and stained with AnnexinV-FITC and propidium iodide, then the apoptosis cells were analyzed by FCM. The apoptotic cells in SKLB-850 group were significantly more than that in R788 and vehicle groups. Columns, mean; bars, SD. ^##^*P<*0.01, or ^#^*P*<0.05, SKLB-850 group versus vehicle group.

Obviously, Syk, Src and JAK2 all are potential targets for BCL treatment. It is reasonable to hypothesize that agents that can simultaneously attack these targets might have enhanced effects on the therapy of BCL. SKLB-850 (Figure 1A) is an orally available multikinase inhibitor potently inhibiting Syk, Src, and JAK2, which was obtained recently by us through a process of computer-aided lead discovery and subsequent structural optimization; the related results will be published elsewhere. The main purpose of this investigation is to perform a comprehensive preclinical evaluation to this compound, including *in vitro* and in *vivo* anti-BCL activity, and mechanism of action.

## RESULTS AND DISCUSSION

### Kinase inhibitory potency of SKLB-850

Kinase inhibitory potency of SKLB-850 against a number of selected kinases were measured with gold-standard^33^ P radio-labeled technology. As shown in Table 1, SKLB-850 is a multikinase inhibitor and potently inhibited Src, Syk, and JAK2 with IC_50_ (half maximal inhibitory concentration) values of 0.025μM, 0.041μM, and 0.047μM, respectively. It also showed some activity against several other kinases, including EGFR (IC_50_: 0.26μM), FAK (IC_50_: 0.39μM), and PKBα (IC_50_: 3.18μM) (Table 1). For the remaining 11 kinases tested, SKLB-850 exibited very weak or no activity (IC_50_ > 10 μM).

**Table 1:** *In vitro* kinase inhibitory activities of SKLB-850 against selected kinases

Biochemical activity	IC_50_ (μM)
**Src**	0.025
**Syk**	0.041
**JAK2**	0.047
**EGRF**	0.255
**FAK**	0.390
**PKBα**	3.183
**CDK3**	>10
**PKCα**	>10
**Pim-1**	>10
**Pim-2**	>10
**GRK1**	>10
**EphB3**	>10
**mTOR**	>10
**PIP4K2α**	>10
**MKK4**	>10
**GSK3α**	>10
**GSK3β**	>10

### *In vitro* activities of anti-tumor cell viability of SKLB-850

*In vitro* anti-viability activities of SKLB-850 against various tumor cell lines were measured using the MTT method. As shown in Table 2, SKLB-850 displayed potent inhibitory activity against human B cell lymphoma cell line Ramos with an IC_50_ value of 0.03 μM. It also showed considerable activities against several other human B cell lymphoma or diffuse large B cell lymphoma cell lines including HBL-1 (IC_50_: 0.69μM), SuDHL-6 (IC_50_: 0.90μM), Ly-10 (IC_50_: 0.90μM), LY-1 (IC_50_: 1.60μM), and RAJI (IC_50_: 1.95μM), and relatively weak activities against solid tumor cell lines MDA-MB-231, SW1990, SMMC7721, MCF-7, and CFPAC-1. To human pancreatic cancer cell line HPAC and human non-small cell lung cancer cell lines A549 and H358, SKLB-850 did not exhibit activity, which excluded the possibility that the anti-viability activity of SKLB-850 is due to cytotoxicity.

**Table 2:** Anti-viability potencies of SKLB-850 against various tumor cell lines.

Cell lines	Cell line type	IC_50_ (μM)
**Ramos-ZHL**	Human B cell lymphoma	0.03
**HBL-1**	Human diffuse large B cell lymphoma	0.69
**SuDHL-6**	Human follicular B cell lymphoma	0.90
**LY-10**	Human diffuse large B cell lymphoma	0.90
**LY-1**	Human diffuse large B cell lymphoma	1.60
**RAJI**	Human B cell lymphoma	1.95
**MDA-MB-231**	Human breast cancer cell	2.19
**SW1990**	Human pancreatic cancer cell	2.19
**SMMC7721**	Human hepatocellular carcinoma cell	2.20
**MCF-7**	Human breast cancer cell	7.23
**CFPAC-1**	Human pancreatic cancer cell	7.24
**HPAC**	Human pancreatic cancer cell	>20
**A549**	Human non-small cell lung cancer cells	>20
**H358**	Human non-small cell lung cancer cells	>20

### SKLB-850 induced apoptosis of tumor cells in *vitro*

The ability of SKLB-850 to induce apoptosis of BCL cells was analyzed by FCM. In this assay, the BCL cell lines Ramos and HBL-1 were selected. As shown in Figure 1B-1E, the percentages of apoptotic cells for both Ramos and HBL-1 cell lines were dose-dependently increased after SKLB-850 treatment for 18 hours. R788, which is a Syk inhibitor and is in clinical trials for the treatment of BCL, also induced apoptosis but obviously much weaker compared with SKLB-850 (Supplementary Figure 1).

### SKLB-850 blocked the tumor cell cycle *in vitro*

**Figure 2:**
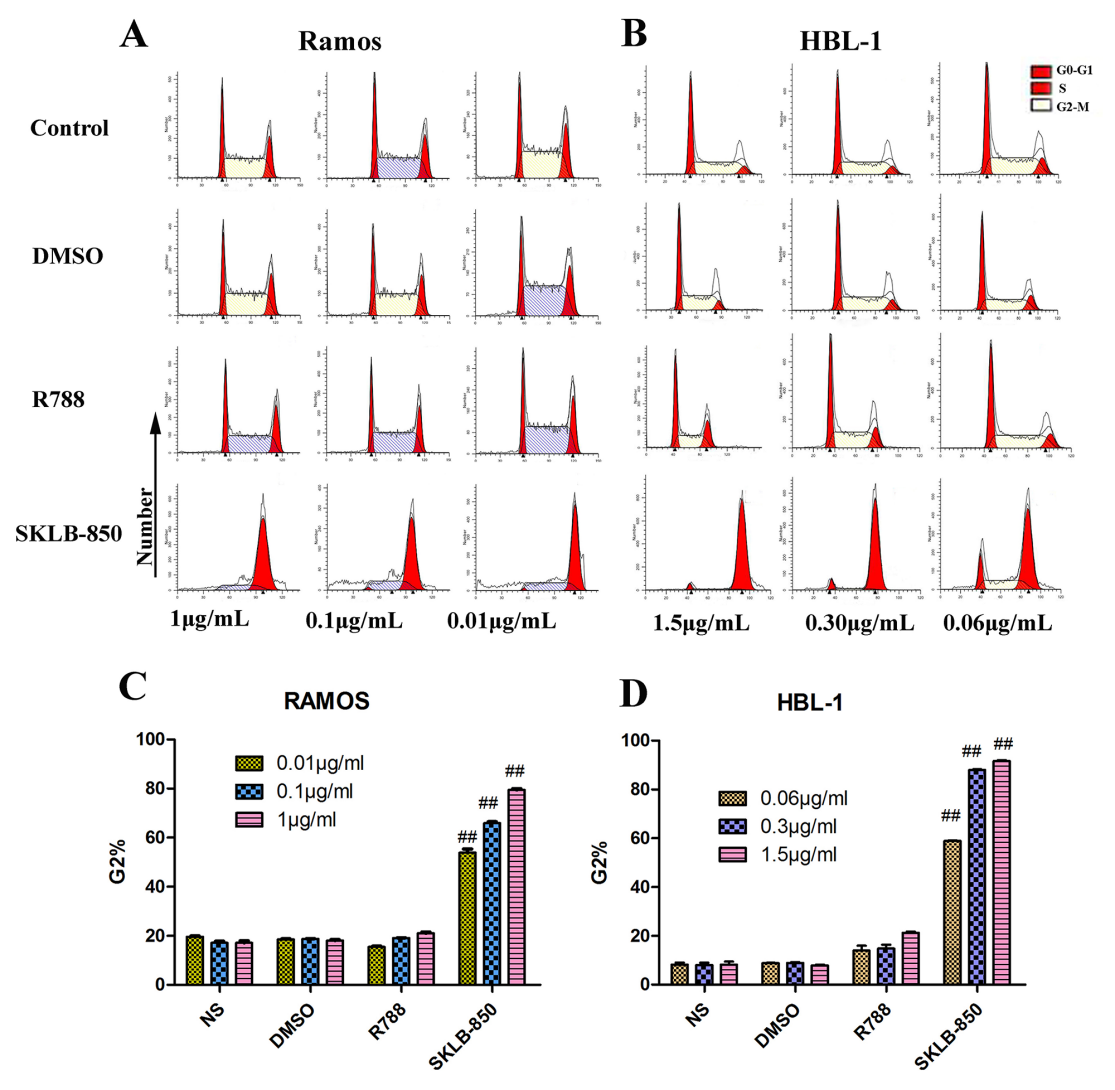
Cell cycle analysis of Ramos and HBL-1 cells induced by SKLB-850, R788 DMSO or control Tumor cells were treated by vehicle, R788 or indicated concentrations of compounds. After 18 hours, cell were harvested and stained with AnnexinV-FITC and propidium iodide, and then analyzed by FCM. The G2 cells in SKLB-850 groups were significantly more than that in R788 and vehicle groups. **(A and C)** Cell cycle analysis of Ramos cells. **(B and D)** Cell cycle analysis of HBL-1 cells. The percent of G2 cells in SKLB-850 groups (*P* < 0.01) was significantly more than that in R788, DMSO and NS groups. Columns, mean; bars, SD.^##^*P* < 0.01, 850 group versus DMSO or blank control group.

FCM was again used to examine the influence of SKLB-850 on the tumor cell cycle. As shown in Figure 2A and 2C, the percentages of Ramos cells in the G2 phase were significantly increased after treatment of SKLB-850 compared with the control. For example, 0.01μg/mL SKLB-850 treatment led to that the percentage of G2 phase cells increased to 53.90 ± 2.65 % (*P*<0.01) from 19.64 ± 0.61 % (*P*<0.01) of the control. Very similarly, the percentages of HBL-1 cells in the G2 phase were also significantly increased after treatment of SKLB-850 compared with the control (see Figure 2B and 2D). In contrast, R788 just had a very weak effect on the percentage of cells in G_2_ phase. All the results indicated that SKLB-850 arrested the Ramos and HBL-1 cells in the G2 phase *in vitro*.

### SKLB-850 inhibited the secretion of chemokines CCL3, CCL4, CXCL12 and expression of CD184

**Figure 3:**
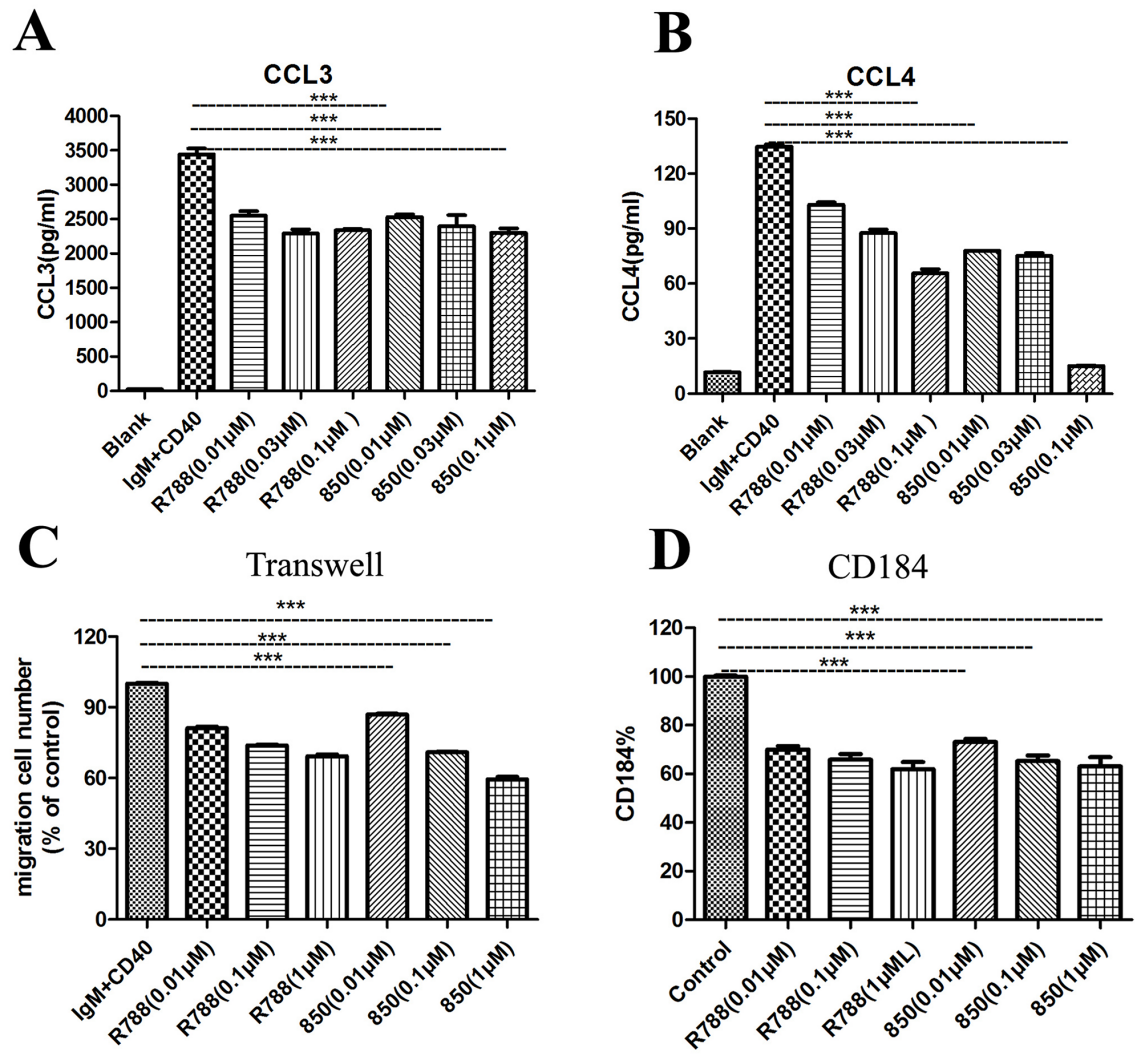
SKLB-850 inhibited anti-IgM and anti-CD40-induced secretion of the chemokines CCL3, CCL4, chemotaxis, and CD184 expression **(A and B)** SKLB-850 inhibited anti-IgM and anti-CD40-induced secretion of the chemokines CCL3, CCL4. **(C)** SKLB-850 inhibited anti-IgM and anti-CD40-induced BCL cell chemotaxis toward the chemokines CXCL12. **(D)** SKLB-850 inhibited anti-IgM and anti-CD40-induced the CD184 expression of Ramos cells. Data represent the mean±SEM. ^**^P<0.01, or ^*^P<0.05, SKLB-850 group versus control group.

We then measured the concentrations of CCL3 and CCL4 in supernatant of Ramos cells cultured in medium, or medium supplemented with anti-IgM and anti-CD40 in the presence or absence of SKLB-850 after 18 hours. CCL3 and CCL4 are chemokines, which appear to correlate with the signaling capacity of the BCR [26]. As shown in Figure 3A and 3B, SKLB-850 inhibited the increased secretion of CCL3 and CCL4 stimulated by anti-IgM and anti-CD40. The mean CCL3 and CCL4 concentrations in supernatants of Ramos cells after anti-IgM and CD40 stimulation were 3443.00 ± 134.80 pg/mL and 134.80 ± 1.90 pg/mL, respectively. Treatment of Ramos cells with 0.1μM SKLB-850 significantly decreased anti-IgM and anti-CD40 induced CCL3 and CCL4 levels to 2303.10 ± 109.40 pg/mL (CCL3, n=3, *P*<0.01) and 15.20 ± 0.30 pg/mL (CCL4, n=3, *P*<0.01), respectively.

Syk has been demonstrated to be involved in the migration of BCL cells. And BCL cells could migrate toward the chemokine CXCL12 upon activation of its receptor CD184 [27]. As displayed in Figure 3C and 3D, SKLB-850 treatment decreased the expression of CD184, and reduced the percentage of Ramos cell chemotaxis toward CXCL12.

### *In vivo* anti-tumor effect of SKLB-850

**Figure 4:**
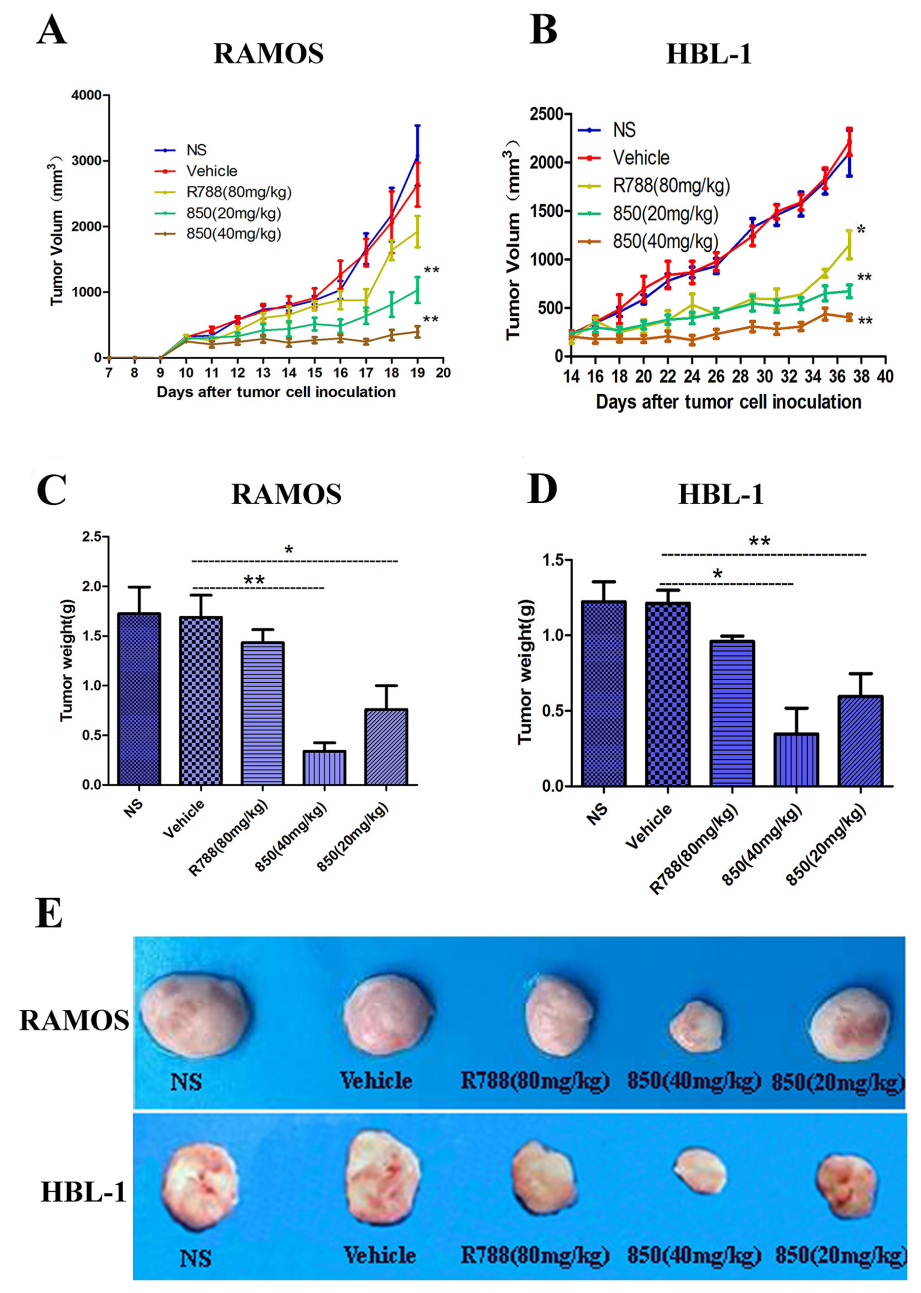
Anti-tumor activities of SKLB-850 *in vivo* **(A and B)** RAMOS or HBL-1 tumor-bearing NOD/SCID mice were treated as described with SKLB-850 at 40 mg/kg or 20 mg/kg, R788 or with vehicle. The treatment with SKLB-850 significantly inhibited tumor growth versus vehicle control (n =6; ANOVA; ^*^P < 0.05, ^**^P < 0.01, R788 or SKLB-850 vs. vehicle control). **(C and D)** The tumor weight of Ramos and HBL-1 tumors with treatment of SKLB-850 or R788, respectively. E: Representative photographs of subcutaneous tumors in Ramos model and HBL-1 model.

The *in vivo* anti-BCL effects of SKLB-850 were investigated in the Ramos and HBL xenograft mouse models with R788 being taken as a positive control. The animals were treated orally once daily for 21 days (Ramos model) or 36 days (HBL-1 model). In the Ramos model, 40 mg/kg or 20 mg/kg SKLB-850 significantly suppressed the tumor growth with tumor inhibition rates of 78.82% ± 9.35% and 73.65% ± 21.22%, respectively (Figure 4A and 4C). Similarly, in the HBL-1 model, SKLB-850 treatment with 40 mg/kg or 20 mg/kg also substantially inhibited the tumor growth with tumor inhibition rates of 67.96% ± 8.40% and 80.84% ±4.09%, respectively (Figure 4B and 4D). In the two models, R788 also showed some anti-tumor effect, but its potencies are relatively weaker than those of SKLB-850.

**Figure 5:**
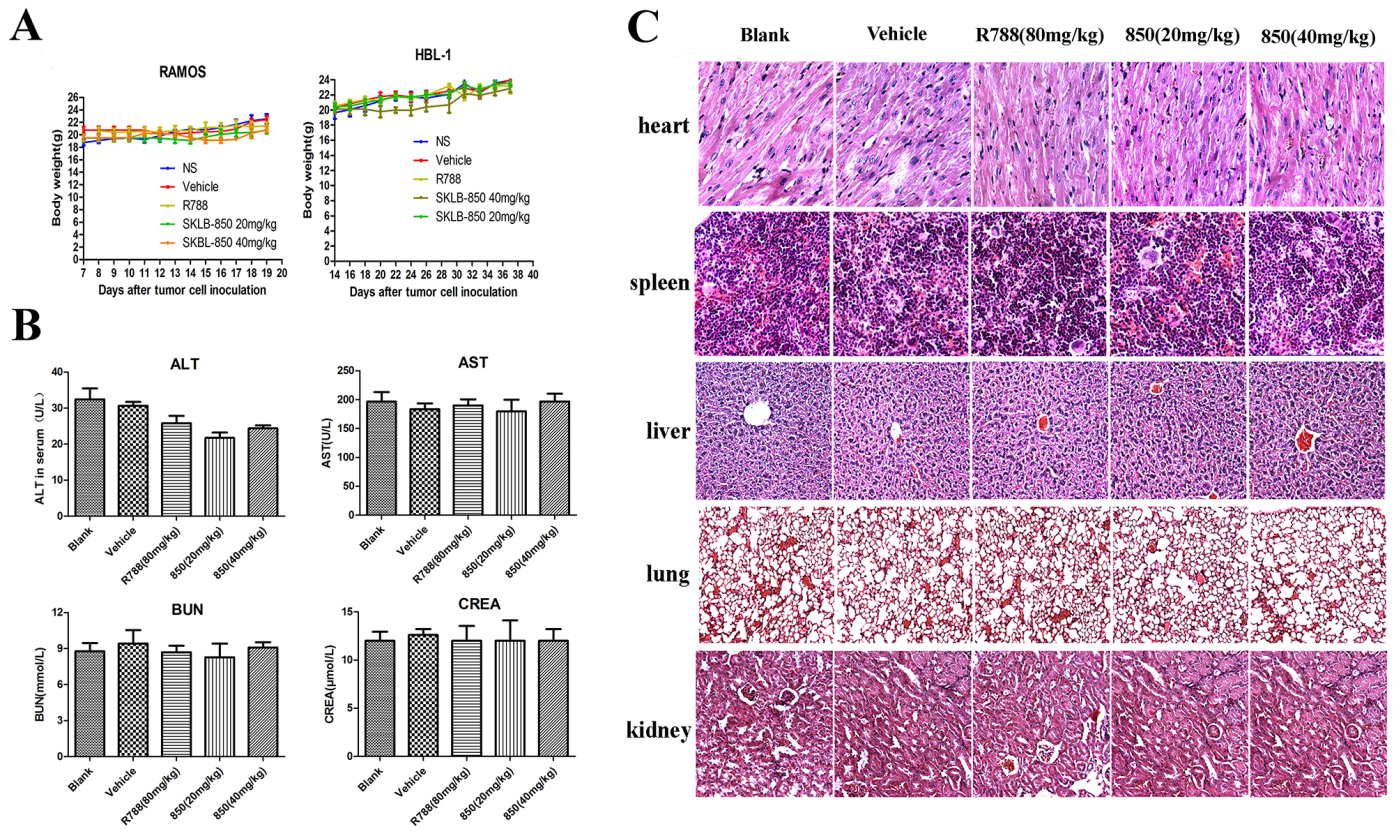
Cytotoxicity evaluation of SKLB-850 **(A)** Mouse body weight with treatment of vehicle, R788, or 850 was monitored once every three days. **(B)** Level of ALT, AST, BUN, and CREA of serum in vehicle, R788 or SKLB-850 group. **(C)** H&E staining of heart, liver, spleen, lung, and kidney tissues in blank, vehicle, R788, or SKLB-850 group.

A preliminary toxicity evaluation to SKLB-850 was also carried out. As shown in Figure 5A, no significant differences in weights were observed for all the treated groups. Furthermore, there were no obvious changes for the levels of ALT, AST, BUN, and CREA, which are important indexes representing liver injury, in mice treated with SKLB-850 (Figure 5B). Besides, toxic pathologic changes in liver, lung, kidney, spleen, and heart were not detected by microscopic examination (Figure 5C). All the results indicated that SKLB-850 had very small systemic toxicity.

### SKLB-850 induced apoptosis in mouse tumor tissues and B-cell lymphoma samples from BCL patients

**Figure 6:**
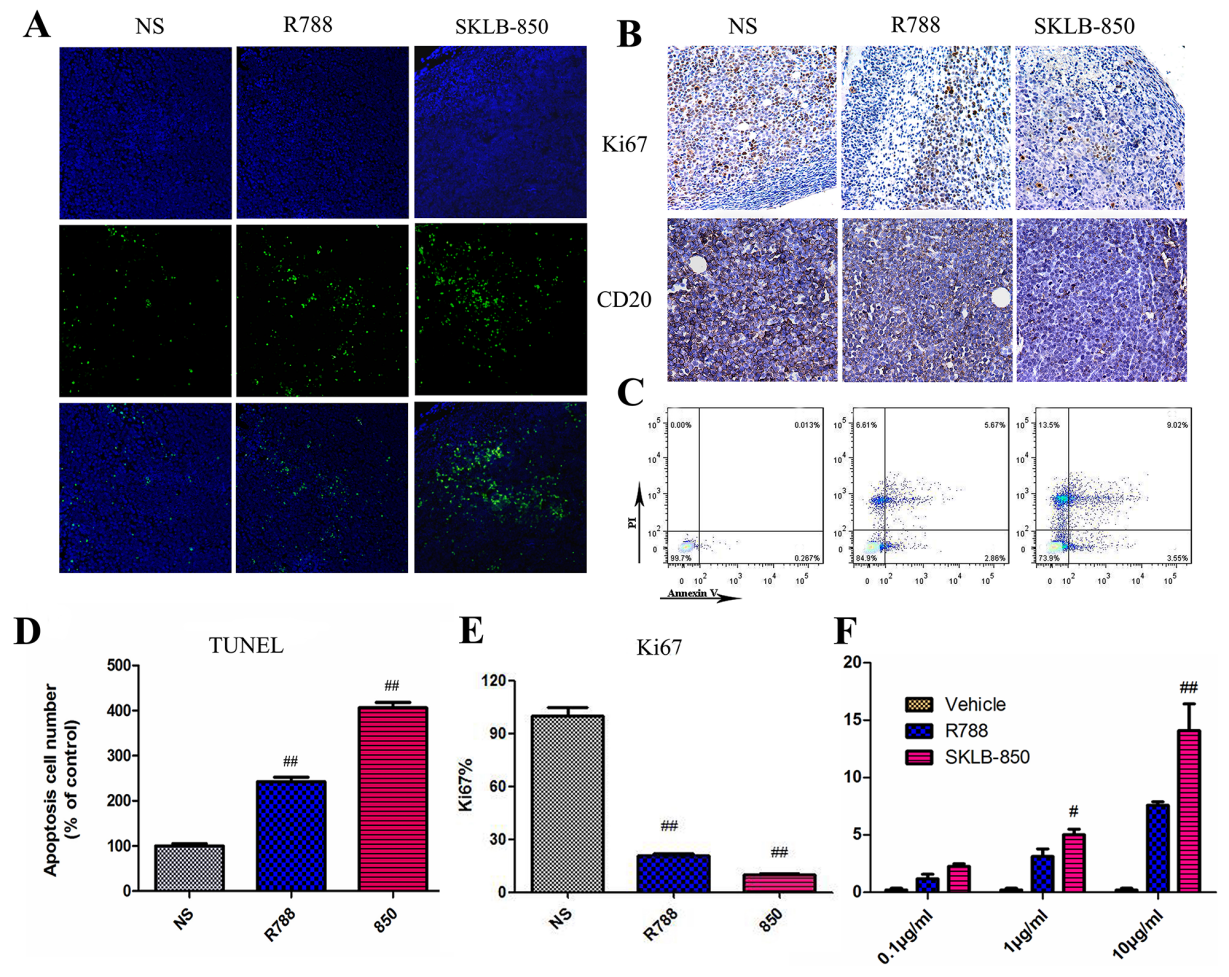
SKLB-850 inhibited tumor growth by inducing apoptosis of tumor tissues from the Ramos model and BCL samples from patients **(A and D)** TUNEL immunofluorescent staining of tumor tissues from the Ramos model (n-6 mice per group). **(B and E)** Immunohistochemical staining analyses of Ki67 and CD20 in tumor tissues from the Ramos model. **(C and F)** Apoptosis of tumor cells from the BCL patients induced by SKLB-850. Tumor tissues from BCL patients were treated with a series of concentrations of SKLB-850 or R788, and stained with AnnexinV-FITC and propidium iodide, then analyzed with a flow cytometer. N=3, Columns, mean; bars, SD. ^##^*P<*0.01, or ^#^*P*<0.05, SKLB-850 group versus vehicle group.

The ability of inducing apoptosis of SKLB-850 in tumor tissues was examined by TUNEL. As shown in Figure 6A and 6D, SKLB-850 significantly induced apoptosis in tumor tissues compared with R788 (SKLB-850, 406.00 ± 20.70% of control; R788, 242.20 ± 20.00% of control). We then examined apoptotic tumor cells in B-cell lymphoma samples from BCL patients after SKLB-850 treatment by FCM. As shown in Figure 6C and 6F, SKLB-850 induced cell apoptosis (14.10 ± 4.04%), and R788 had the same effect but relatively weak (7.60 ± 0.52%). To better understand the mechanism of antitumor activities *in vivo*, Ki67 and CD20 in mouse tumor tissues were detected by immunohischemistry; Ki67 was a marker of proliferation in the diagnosis of tumors and CD20 was highly expressed in B cell lymphomas [28]. We observed significant reduction of Ki67 and CD20 expression when treated by SKLB-850, indicating inhibition activities of SKLB-850 in tumor tissues (Figure 6B and 6E). All the data showed that SKLB-850 could induce apoptosis in *vivo* and B lymphoma cells of patients, decrease expression of Ki67 and CD20, which contribute to the tumor growth suppression.

### SKLB-850 inhibited tumor growth via blocking Syk/ERK, Src/FAK and JAK2/STAT3 signaling pathways

**Figure 7:**
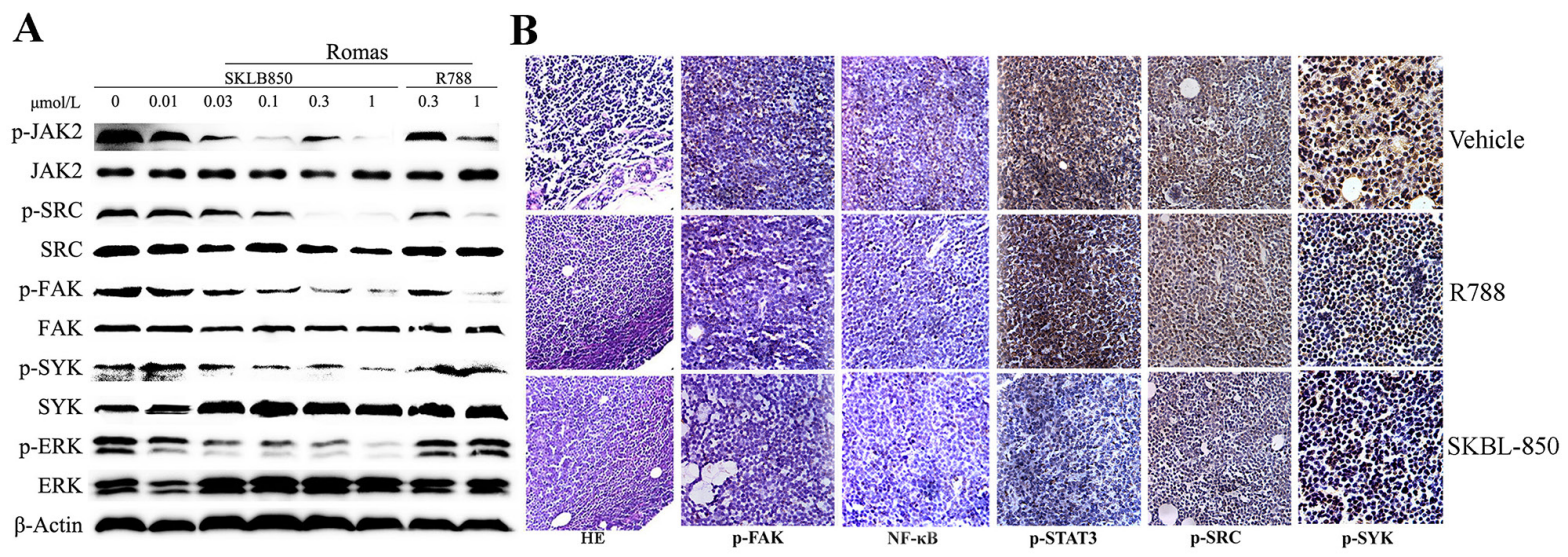
Western blot and histochemical analyses of tumor tissues from the Ramos model **(A)** SKLB-850 inhibited Syk/ERK, Src/FAK and JAK2/STAT3 signaling pathways. The Ramos cells were incubated for 24 hours in medium containing vehicle, SKLB-850, or R788, and then lysed for western blot assay. **(B)** H&E staining and immunohistochemical staining of NF-κB, p-Src, p-STAT3 and p-Syk in tumor tissues isolated from vehicle, R788 or SKLB-850 treated groups. SKBL-850 inhibited p-JAK2, p-FAK, p-Src, p-ERK and p-Syk expression in RAMOS cells.

To understand the mechanism of antitumor activities in *vivo*, pivotal protein activities of Syk/ERK, Src/FAK and JAK2/STAT3 signaling pathways were detected by Western blot analysis. In Figure 7A and 7B, SKLB-850 effectively inhibited Syk, Src, JAK2 phosphorylation, and down-regulated the phosphorylation levels of ERK, FAK and Stat3 at concentrations between 0.01μMl and 1μM/ml in Ramos cell. These results imply that mechanisms of anti-tumor action were related with inhibition of Syk/ERK, Src/FAK and JAK2/STAT3 signaling pathways. Immunohistochemistry assays were performed using tumor tissues isolated from the Ramos tumor model at the end of treatment. As shown in Figure 7B, phosphorylations of Syk, Src/FAK and JAK2/STAT3 were inhibited in SKLB-850 treatment group. In addition, phosphorylation of NF-κB p65, one of the proteins in Syk-regulated signaling pathways, was also inhibited by SKLB-850 in tumor tissues. In conclusion, SKLB-850 inhibited tumor growth by blocking Syk/ERK, Src/FAK and JAK2/STAT3 signaling pathways (Figure 7).

## MATERIAL AND METHODS

### Cell lines

Human cancer cell lines Ramos-ZHL, HBL-1, SuDHL-6, LY-10, LY-1, RAJI, MDA-MB-231, SW1990, SMMC7721, MCF-7, CFPAC-1, HPAC, A549 and H358 were obtained from American Type Culture Collection (ATCC). All tumor cell lines were maintained according to the ATCC procedures and passaged for less than 6 months after receipt for resuscitation. These cells were cultured in RPMI-1640 supplemented with 10% fetal bovine serum (FBS).

### Kinase inhibition assay

The IC_50_ values of SKLB-850 for kinase inhibition *in vitro* were measured by radiometric assays conducted by Kinase Profiler service provided by Millipore as described in Pan’s study [29].

### Preparation of SKLB-850

SKLB-850 (5-chloro-2-((5-fluoro-2-((4-(4-methyl piperazin-1-yl) phenyl) amino) pyrimidin-4-yl) amino) benzamide) was synthesized at the State Key Laboratory of Biotherapy, Sichuan University. For all *in vitro* assays, SKLB-850 was prepared initially as a 10mg/mL stock solution in dimethylsulfoxide (DMSO). Stock solution was diluted in the relevant assay media, and 0.1% DMSO served as a vehicle control. For studies in athymic mice, SKLB-850 was suspended in 25% (v/v) polyethylene glycol solution containing 5% (v/v) DMSO and dosed at 0.1 mL/10g of body weight.

### Cell proliferation assay

Cell proliferation was measured using the MTT assay as previously described [30]. Various cells including were treated with indicated concentrations of SKLB-850 for 72 hours. R788 (Selleck.cn) was served as a positive control. Each assay was replicated 3 times.

### Apoptosis assay

Flow cytometry (FCM) assays were performed to investigate the apoptosis of Ramos and HBL-1 cells induced by SKLB-850. Ramos and HBL-1 cell cultures in plates were treated with indicated concentrations of SKLB-850 or R788 for 24 hours. Medium without treatment reagents were added as control. Cells were harvested and washed with PBS, stained with 2.5μL AnnexinV-FITC and 2.5μL propidium iodide (Beyotime Institute of Biotechnology). Cell apoptosis was analyzed with a flow cytometer (BD FACSCalibur, BD, USA).

### Cell cycle analysis

Ramos and HBL-1 cells cultured in 6-well plates were treated with DMSO, R788, SKLB-850 or medium. After 18 hours, cells were harvested and washed with PBS, fixed with 70% ethanol for 30 min, stained with 200 μL PI (50 μg/mL PI with 1 mg/mL RNase I, and 0.1% Triton X-100) for 30 min. Cell cycle was analyzed with a flow cytometer (BD FACSCalibur, BD, USA).

### Enzyme-linked immunosorbent assays

To evaluate the effect of SKLB-850 on CCL3 and CCL4 secretion after stimulation of Ramos cells with anti-IgM (30 μg/ml) and anti-CD40 (10 μg/ml), chemokine levels were measured in supernatants of activated BCL cells, as previously described [31]. After 18 hours of anti-IgM and anti-CD40 co-culture, supernatants were harvested and assayed for CCL3 and CCL4 by quantitative enzyme-linked immunosorbent assays according to the manufacturer’s instructions (Quantikine, R&D Systems, Minneapolis, MN, USA).

Chemotaxis assay was performed as previously described [31]. Briefly, 1×10^6^ Ramos cells were cultured in RPMI 1640 containing 0.5% bovine serum albumin and were pre-incubated for 30 minutes with SKLB-850, R788 or DMSO, and then they were added to the inserts of transwell chambers (5μm poresize; Corning). Inserts were then transferred into wells containing CXCL12 (200ng/mL). After 12 hours, cell count was determined for the upper and lower well in duplicates using a flow cytometer (Novocyte Technologies, China). Chemotaxis indices were calculated as: [number of cells in the lower chamber×100] / [number of cells in the lower and upper chambers]. The chemotaxis index with SKLB-850 was calculated as percentage of the DMSO index.

### Flow cytometry

The expression of surface molecules was analyzed by flow cytometry, using the following monoclonal antibody CD184 (CXCR4), and the relevant isotype controls mAbs (BD biosciences). A total of 10^6^ mononuclear cells were incubated for 30 minutes at 4°C with saturating concentrations of mAbs, and the cells were washed twice and analyzed with a flow cytometer (BD FACSCalibur, BD, USA).

### Western blot analysis

For Ramos cell immunoplot studies, cancer cells were incubated with vehicle, R788, or SKLB-850 for 24 hours, and then lysed in RIPA buffer (Beyotime, China) containing Roche protease inhibitor cocktail, and the protein concentrations were determined by the Bradford method. Proteins were separated by gel electrophoresis on 8–12% SDS-PAGE gels and probed with specific antibodies (Cell Signaling Technology, USA) including anti-Caspase3, anti-FAK, anti-pFAK^Tyr925^, anti-Src, anti-pSrc^Tyr416^, anti-Syk, anti-pSyk, anti-ERK, anti-pERK^Thr202/Tyr204^ and anti-β-actin. Blots were developed with horseradish peroxidase (HRP)-conjugated secondary antibodies (Zhong Shan Golden Bridge Biotechnology, China) and chemiluminescent substrate on Kodak X-ray films.

### Xenograft mouse model

All animal studies were conducted according to the guidelines of the Animal Care and Use Committee of Sichuan University (Permit Number: 20121205, Chengdu, Sichuan, China). Ramos and HBL-1 tumor cells were established by subcutaneously injecting 1×10^7^ cells (100μL) into the hind flank region of 7–8 week old female NOD/SCID mice (ICR, NOD/SCID). When tumors reached a volume of ∼200 mm^3^, mice were randomly divided into different groups (6 per group), and SKLB-850 (40 mg/kg, 20mg/kg) or vehicle (5% DMSO, 25% PEG400, and 70% waters) were given once daily by oral gavage. Tumor growth and body weight were measured every three days or every day during the treatment. Tumor volumes were calculated using the formula as follow: tumor volume (mm^3^) = 0.5× length (mm) × width (mm) ^2^. Studies were typically terminated when tumors in vehicle treated animals reached an average size of 2,000 mm^3^. Solid tumors were removed and processed for immunohistochemical analysis and terminal deoxynucleotidyl transferase-mediated dUTP nick end labeling (TUNEL) assay.

### Histopathology and immunohistochemistry

Paraffin sections from each group were stained with hematoxylineosin (H&E). For immunohistochemistry studies, tumor tissues in the Ramos model were performed and the following antibodies were used: phospho-Src (Cell Signaling Technology, 1:100), phospho-FAK (Cell Signaling Technology, 1:100), phospho-Stat3 (Cell Signaling Technology, 1:100), NF-κB p-65(Cell Signaling Technology, 1:100) and phospho-Syk (Cell Signaling Technology, 1:100). Additionally, Rat anti-mouse CD31 antibody (BD Biosciences, USA) and anti-Ki67 antibody (Gene Tech) were used to determine vessel density and cell proliferation following the manufacturer’s protocol, respectively. A DAKO polymer secondary antibody system (Dako Envision 1K4007) was used for secondary detection. Images were captured using an Olympus digital camera.

### *In situ* TUNEL

Cell apoptosis in Ramos tumors was determined by a TUNEL assay following the manufacturer’s instructions (Promega). The number of TUNEL-positive cells was quantified by fluorescence microscopy and the apoptotic index in 6 random fields per group was counted.

### Toxicity evaluation

To investigate potential side effects or toxicity on mice during the treatment, anorexia skin ulceration, weight loss, diarrhea, and toxic death were observed continuously for relevant indexes. The tissues of heart, liver, spleen, lung, and kidney were stained with hematoxylin and eosin (H&E). ALT, AST, BUN and CREA in serum were measured by the National Chengdu Center for Safety Evaluation of Traditional Chinese Medicine using commercially available kits.

### SKLB-850 induced apoptosis of human B cell lymphoma from patients

Human lymph nodes were collected aseptically from B-cell lymphoma patients (n = 4 for both groups, Supplementary Table 1). For the investigation of apoptosis induced by SKLB-850, human lymph nodes tissues were minced into ∼1mm^3^ cubes, washed it with HBSS, and cultured with vehicle, R788, or SKLB-850 (10μg/ml, 1μg/ml, 0.1μg/ml) at 37°C for 24 hours. Tumor cells were harvested and washed with PBS, stained with 2μL AnnexinV-FITC and 2μL propidium iodide (Beyotime Institute of Biotechnology). Cell apoptosis was analyzed with a flow cytometer (BD FACS Calibur, BD, USA).

### Statistical analysis

The statistical analysis was carried out using SPSS 17.0 software (Chicago, IL, USA). Data were presented as means ± SD and analyzed statistically by using one-way ANOVA followed by the Turkey’s test. Values of *P* < 0.05 were indicative of significant differences and *P* < 0.01 was indicative of a very significant difference.

## CONCLUSIONS

In summary, preclinical anti-BCL activities and mechanisms of action of SKLB-850 were investigated. SKLB-850 is a multikinase inhibitor and potently inhibited Syk, Src and JAK2 with IC_50_ values of 0.041μM, 0.025μM and 0.047μM, respectively. In *in vitro* anti-viability assays, SKLB-850 showed considerable anti-viability potencies against human BCL cell lines Ramos and HBL-1. It could significantly induce BCL cell apoptosis, and arrested the cell cycle in the G2 phase. Furthermore, SKLB-850 inhibited anti-IgM and anti-CD40-induced secretion of CCL3, CCL4 and CXCL12. In Ramos and HBL xenograft mouse models, SKLB-850 displayed potent anti-tumor activities. Immunohistochemistry analysis showed that SKLB-850 inhibited expression of Ki67 and CD20 and down-regulated activities of Syk/ERK, Src/FAK and JAK2/STAT3 signaling pathways. Collectively, SKLB-850 could be a promising agent for the treatment of BCL, hence deserving further study.

## References

[1] Muller AM, Ihorst G, Mertelsmann R, Engelhardt M (2005). Epidemiology of non-Hodgkin’s lymphoma (NHL): trends, geographic distribution, and etiology. Ann Hematol.

[2] Tuccori M, Focosi D, Blandizzi C, Pelosini M, Montagnani S, Maggi F, Pistello M, Antonioli L, Fornai M, Pepe P, Rossi G, Petrini M (2010). Inclusion of rituximab in treatment protocols for non-Hodgkin’s lymphomas and risk for progressive multifocal leukoencephalopathy. Oncologist.

[3] Alinari L, Quinion C, Blum KA (2015). Bruton’s tyrosine kinase inhibitors in B-cell non-Hodgkin’s lymphomas. Clin Pharmacol Ther.

[4] Dotan E, Aggarwal C, Smith MR (2010). Impact of Rituximab (Rituxan) on the treatment of B-cell non-Hodgkin’s lymphoma. P T.

[5] Ram R, Ben-Bassat I, Shpilberg O, Polliack A, Raanani P (2009). The late adverse events of rituximab therapy--rare but there!. Leuk Lymphoma.

[6] Arai Y, Yamashita K, Mizugishi K, Nishikori M, Hishizawa M, Kondo T, Kitano T, Kawabata H, Kadowaki N, Takaori-Kondo A (2015). Risk factors for late-onset neutropenia after rituximab treatment of B-cell lymphoma. Hematology.

[7] Zhang SQ, Smith SM, Zhang SY, Lynn Wang Y (2015). Mechanisms of ibrutinib resistance in chronic lymphocytic leukaemia and non-Hodgkin lymphoma. Br J Haematol.

[8] Sinha G (2014). Overcoming mantle cell lymphoma’s ibrutinib resistance. J Natl Cancer Inst.

[9] Quiroga MP, Balakrishnan K, Kurtova AV, Sivina M, Keating MJ, Wierda WG, Gandhi V, Burger JA (2009). B-cell antigen receptor signaling enhances chronic lymphocytic leukemia cell migration and survival: specific targeting with a novel spleen tyrosine kinase inhibitor, R406. Blood.

[10] Suljagic M, Longo PG, Bennardo S, Perlas E, Leone G, Laurenti L, Efremov DG (2010). The Syk inhibitor fostamatinib disodium (R788) inhibits tumor growth in the Emu- TCL1 transgenic mouse model of CLL by blocking antigen-dependent B-cell receptor signaling. Blood.

[11] Burger JA, Chiorazzi N (2013). B cell receptor signaling in chronic lymphocytic leukemia. Trends Immunol.

[12] Sharman J, Hawkins M, Kolibaba K, Boxer M, Klein L, Wu M, Hu J, Abella S, Yasenchak C (2015). An open-label phase 2 trial of entospletinib (GS-9973), a selective spleen tyrosine kinase inhibitor, in chronic lymphocytic leukemia. Blood.

[13] Young RM, Hardy IR, Clarke RL, Lundy N, Pine P, Turner BC, Potter TA, Refaeli Y (2009). Mouse models of non-Hodgkin lymphoma reveal Syk as an important therapeutic target. Blood.

[14] Friedberg JW, Sharman J, Sweetenham J, Johnston PB, Vose JM, Lacasce A, Schaefer-Cutillo J, De Vos S, Sinha R, Leonard JP, Cripe LD, Gregory SA, Sterba MP (2010). Inhibition of Syk with fostamatinib disodium has significant clinical activity in non-Hodgkin lymphoma and chronic lymphocytic leukemia. Blood.

[15] Szydlowski M, Kiliszek P, Sewastianik T, Jablonska E, Bialopiotrowicz E, Gorniak P, Polak A, Markowicz S, Nowak E, Grygorowicz MA, Prochorec-Sobieszek M, Szumera-Cieckiewicz A, Malenda A (2016). FOXO1 activation is an effector of SYK and AKT inhibition in tonic BCR signal-dependent diffuse large B-cell lymphomas. Blood.

[16] Sen B, Johnson FM (2011). Regulation of SRC family kinases in human cancers. J Signal Transduct.

[17] Peiro G, Ortiz-Martinez F, Gallardo A, Perez-Balaguer A, Sanchez-Paya J, Ponce JJ, Tibau A, Lopez-Vilaro L, Escuin D, Adrover E, Barnadas A, Lerma E (2014). Src, a potential target for overcoming trastuzumab resistance in HER2-positive breast carcinoma. Br J Cancer.

[18] Vazquez-Franco JE, Reyes-Maldonado E, Vela-Ojeda J, Dominguez-Lopez ML, Lezama RA (2012). Src, Akt, NF-kappaB, BCL-2 and c-IAP1 may be involved in an anti-apoptotic effect in patients with BCR-ABL positive and BCR-ABL negative acute lymphoblastic leukemia. Leuk Res.

[19] Ke J, Chelvarajan RL, Sindhava V, Robertson DA, Lekakis L, Jennings CD, Bondada S (2009). Anomalous constitutive Src kinase activity promotes B lymphoma survival and growth. Mol Cancer.

[20] Hatton O, Lambert SL, Krams SM, Martinez OM (2012). Src kinase and Syk activation initiate PI3K signaling by a chimeric latent membrane protein 1 in Epstein-Barr virus (EBV)+ B cell lymphomas. PLoS One.

[21] Ho K, Valdez F, Garcia R, Tirado CA (2010). JAK2 translocations in hematological malignancies: Review of the literature. J Assoc Genet Technol.

[22] Gupta M, Han JJ, Stenson M, Maurer M, Wellik L, Hu G, Ziesmer S, Dogan A, Witzig TE (2012). Elevated serum IL-10 levels in diffuse large B-cell lymphoma: a mechanism of aberrant JAK2 activation. Blood.

[23] Malinge S, Ben-Abdelali R, Settegrana C, Radford-Weiss I, Debre M, Beldjord K, Macintyre EA, Villeval JL, Vainchenker W, Berger R, Bernard OA, Delabesse E, Penard-Lacronique V (2007). Novel activating JAK2 mutation in a patient with down syndrome and B-cell precursor acute lymphoblastic leukemia. Blood.

[24] Poulain S, Merchez M, Daudignon A, Simon M, Duthilleul P, Morel P (2006). JAK2 V617F mutation is absent in chronic lymphocytic leukemia. Leuk Lymphoma.

[25] Hao Y, Chapuy B, Monti S, Sun HH, Rodig SJ, Shipp MA (2014). Selective JAK2 inhibition specifically decreases Hodgkin lymphoma and mediastinal large B-cell lymphoma growth *in vitro* and *in vivo*. Clin Cancer Res.

[26] Sivina M, Hartmann E, Kipps TJ, Rassenti L, Krupnik D, Lerner S, LaPushin R, Xiao L, Huang X, Werner L, Neuberg D, Kantarjian H, O’Brien S (2011). CCL3 (MIP-1alpha) plasma levels and the risk for disease progression in chronic lymphocytic leukemia. Blood.

[27] Randhawa S, Cho BS, Ghosh D, Sivina M, Koehrer S, Muschen M, Peled A, Davis RE, Konopleva M, Burger JA (2016). Effects of pharmacological and genetic disruption of CXCR4 chemokine receptor function in B-cell acute lymphoblastic leukaemia. Br J Haematol.

[28] Nevala WK, Butterfield JT, Sutor SL, Knauer DJ, Markovic SN (2017). Antibody-targeted paclitaxel loaded nanoparticles for the treatment of CD20+ B-cell lymphoma. Sci Rep.

[29] Pan Y, Xu Y, Feng S, Luo S, Zheng R, Yang J, Wang L, Zhong L, Yang HY, Wang BL, Yu Y, Liu J, Cao Z (2012). SKLB1206, a novel orally available multikinase inhibitor targeting EGFR activating and T790M mutants, ErbB2, ErbB4, and VEGFR2, displays potent antitumor activity both *in vitro* and *in vivo*. Mol Cancer Ther.

[30] Zhang S, Cao Z, Tian H, Shen G, Ma Y, Xie H, Liu Y, Zhao C, Deng S, Yang Y, Zheng R, Li W, Zhang N (2011). SKLB1002, a novel potent inhibitor of VEGF receptor 2 signaling, inhibits angiogenesis and tumor growth *in vivo*. Clin Cancer Res.

[31] Hoellenriegel J, Coffey GP, Sinha U, Pandey A, Sivina M, Ferrajoli A, Ravandi F, Wierda WG, O’Brien S, Keating MJ, Burger JA (2012). Selective, novel spleen tyrosine kinase (Syk) inhibitors suppress chronic lymphocytic leukemia B-cell activation and migration. Leukemia.

