# Supplementary material for: A novel orally available Syk/Src/Jak2 inhibitor, SKLB-850, showed potent anti-tumor activities in B cell lymphoma (BCL) models
Source: Oncotarget. 2017 Dec 1;8(67):111495–507. doi: 10.18632/oncotarget.22847 (PMC5762338; doi:10.18632/oncotarget.22847)
Supplement: Supplementary file 1 [file oncotarget-08-111495-s001.pdf]

A novel orally available Syk/Src/Jak2 inhibitor, SKLB-850, showed potent anti-tumor activities in B cell lymphoma (BCL) models

SUPPLEMENTARY MATERIALS

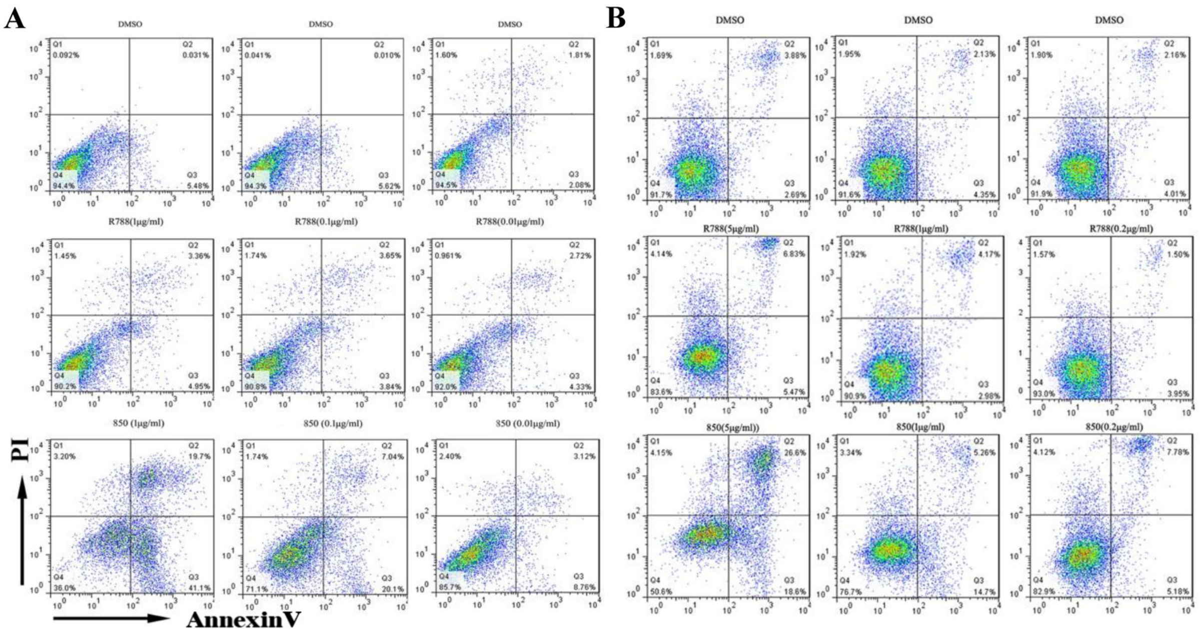

Supplementary Figure 1: SKLB-850 induced tumor cell apoptosis *in vitro*. (A) Apoptosis of Ramos cells induced by SKLB-850. **(B)** Apoptosis of HBL-1 cells induced by SKLB-850. Cells were treated vehicle, R788 or indicated concentrations of compounds. After 18 hours, Cell were harvested and stained with AnnexinV-FITC and propidium iodide, then the apoptosis cells were analyzed by FCM.

Supplementary Table 1: Patient characteristics

Age	Sex	Disease
**66**	female	Diffuse small B lymphoma
**22**	female	follicular lymphoma
**47**	male	Chronic Lymphocytic Leukemia
**40**	male	Diffuse large B lymphoma
**63**	female	Diffuse large B lymphoma
